# Spatial chaos and complexity in the intracellular space of cancer and normal cells

**DOI:** 10.1186/1742-4682-10-62

**Published:** 2013-10-24

**Authors:** Tuan D Pham, Kazuhisa Ichikawa

**Affiliations:** 1 Aizu Research Cluster for Medical Engineering and Informatics, Center for Advanced Information Science and Technology, The University of Aizu, 965-8580, Aizuwakamatsu, Fukushima, Japan; 2 Division of Mathematical Oncology, The Institute of Medical Science, The University of Tokyo, 108-8639, Tokyo, Japan

**Keywords:** Cancer and normal cells, Intracellular space, Bioimaging, Chaos, Nonlinear dynamics

## Abstract

**Background:**

One of the most challenging problems in biological image analysis is the quantification of the dynamical mechanism and complexity of the intracellular space. This paper investigates potential spatial chaos and complex behavior of the intracellular space of typical cancer and normal cell images whose structural details are revealed by the combination of scanning electron microscopy and focused ion beam systems. Such numerical quantifications have important implications for computer modeling and simulation of diseases.

**Methods:**

Cancer cell lines derived from a human head and neck squamous cell carcinoma (SCC-61) and normal mouse embryonic fibroblast (MEF) cells produced by focused ion beam scanning electron microscopes were used in this study. Spatial distributions of the organelles of cancer and normal cells can be analyzed at both short range and long range of the bounded dynamical system of the image space, depending on the orientations of the spatial cell. A procedure was designed for calculating the largest Lyapunov exponent, which is an indicator of the potential chaotic behavior in intracellular images. Furthermore, the sample entropy and regularity dimension were applied to measure the complexity of the intracellular images.

**Results:**

Positive values of the largest Lyapunov exponents (LLEs) of the intracellular space of the SCC-61 were obtained in different spatial orientations for both long-range and short-range models, suggesting the chaotic behavior of the cell. The MEF has smaller positive values of LLEs in the long range than those of the SCC-61, and zero vales of the LLEs in the short range analysis, suggesting a non-chaotic behavior. The intracellular space of the SCC-61 is found to be more complex than that of the MEF. The degree of complexity measured in the spatial distribution of the intracellular space in the diagonal direction was found to be approximately twice larger than the complexity measured in the horizontal and vertical directions.

**Conclusion:**

Initial findings are promising for characterizing different types of cells and therefore useful for studying cancer cells in the spatial domain using state-of-the-art imaging technology. The measures of the chaotic behavior and complexity of the spatial cell will help computational biologists gain insights into identifying associations between the oscillation patterns and spatial parameters of cells, and appropriate model for simulating cancer cell signaling networks for cancer treatment and new drug discovery.

## Introduction

It has been recently reported that cell shape, subcellular sizes, and spatial intracellular distribution can control how molecules interact to produce a cellular behavior [1]. Such spatial information of the cell is important and considered as underlying mechanism for mathematical modeling of cell signaling. In the study of cell plasticity, which refers to the ability of cells to take on the characteristics of other cells in an organism (for example, bone marrow stem cells that are transplanted elsewhere can change into lung or liver cells for repairing damages to internal organs), it is critical to understand the dynamical behavior of the molecular processes in single cells underlying cell plasticity by computational methods that implement experimental parameters of the intracellular single-cell space into the mathematical simulation of complex molecular processes [2].

In fact the construction of a good spatial model of the cell has been suggested to be necessary for studying both deterministic and stochastic phenomena of molecular dynamics [3-5].

Furthermore, it has been observed that the spatial distributions of protein localization within a single cell are dynamic. For example, the surface distribution and dynamic movement of the localization of an outer membrane protein in *Escherichia coli* were found to be complex and changing rapidly [6].

All of these intercellular properties are important for cell simulation which is aimed to be able to predict cell behavior [7] as well as to construct biochemical reactions and electrophysiological models of neurons and other cell types [8].

In the study of cellular dynamics, the hypothesis of cancer as a chaotic process is the most prominent among multiple hypotheses of cancerogenesis [9,10]. Indeed, the idea of adopting chaos for modeling cancer process has been investigated including studies in genetics [10,11], extracellular [12-19] and intracellular [20,21] phenomena of tumor growth. However, these models were mostly constructed based on experience and experiments. In particular, at intracellular level [20-23], the spatial distribution and shape of organelles is still not utilized for studying the possible chaotic behavior of cancer. While many hypotheses and findings about complex patterns of cells, particularly the dynamics of cancer cells associated with chaos, have been reported in literature, there is rarely any work exploring the irregularity and heterogeneity of spatial distribution of cancer intracellular space in images. Such a theoretical study will be helpful to help scientists gain insights into how cells utilize intracellular features to optimize their signaling characteristics [24] and to identify appropriate strategies for modeling and simulation of cancer cellular signaling pathways [25].

In this study, we are particularly interested in the analysis of the spatial chaos and complexity of the intracellular information of cancer and normal cells using the combination of scanning electron microscopy (SEM), which is well known for capturing surface characteristics and morphology, and focused ion beam (FIB), which is increasingly used in biology for site-specific analysis [26].

In fact, the identification of complexity and chaotic behavior in medical and biological systems has been an active area of research [27,28]. On the other hand, evidence for deterministic chaos has been reported from various experiments on nonlinear systems that has given rise to the need for detecting and quantifying chaos [29]. In chaos theory, the notion of Lyapunov exponents is one of the most popular methods for quantifying chaos [30-32] as it was proven to be the most useful tool for studying chaotic systems [33]. Because the computation of the full Lyapunov spectrum is difficult and the estimate of the largest Lyapunov exponent (LLE) is preferred for identifying chaos, many methods have been developed for estimating the LLE. One of the most practical and reliable methods for the computation of the LLE, including small datasets, is the algorithm proposed by Rosenstein *et al.*[34].

Regarding the complexity analysis of signals in terms of sequential irregularity, approximate entropy (ApEn) [35] can quantify such complexity by assessing the randomness of the system. ApEn has the following interesting properties [36]: (1) it is a combinatorial algorithm which can avoid failure in a number of settings for specified sequences, (2) it can be applied to sequences of both finite and infinite length, and (3) its computation is explicit, in counterpoint to algorithmic complexity and axiomatic probability theory. A low value of ApEn indicates that the signal is deterministic and of low complexity. A high value of ApEn implies that the signal is subject to a high degree of complexity and therefore difficult to predict. Sample entropy (SampEn) [28] was developed to avoid bias encountered in ApEn by excluding the counting of a self-match for each sub-sequence of the signal. Both ApEn and SampEn estimate the probability that the sequences in a dataset which are initially closely related remain closely related, within a given similarity tolerance, on the next incremental comparison. However, both methods require a priori knowledge of two unknown parameters *m* (sub-sequence length) and *r* (similarity tolerance), which can be difficult to determine. The regularity dimension (RD) [37] has recently been introduced to overcome such difficulty by allowing the specification of a range of possible values of these two critical parameters *m* and *r* instead of using single values of these two parameters.

The contributions of this paper are that (1) it presents the first attempt in quantifying the spatial dynamics of FIB-SEM-based intracellular space of cancer cells, (2) it introduces the first study on complexity analysis of FIB-SEM-based intracellular space of cancer cells, and (3) it develops original procedures for estimating the largest Lyapunov exponents and regularity dimension of images. Such quantifications of the dynamical behavior and complexity of cancer cells will help life-science researchers gain important information about spatial cell structures based on which effective strategies for cancer modeling and simulation can be established.

The rest of this paper is organized as follows. Section “Materials and methods for sample preparation” presents a proposed procedure for calculating the largest Lyapunov exponents which is used as quantitative measure of potential chaos in intracellular images. Section “Largest Lyapunov exponents of images” introduces the regularity dimension as quantitative measure of complexity applied to images. Section “Regularity dimension of images” includes experimental results and discussion. Finally, Section “Results” is the conclusion of the new findings.

## Materials and methods for sample preparation

We used two types of cultured cells in this study: cancer cell lines derived from a human head and neck squamous cell carcinoma (SCC-61) parental line [38,39], and normal cells of the mouse embryonic fibroblasts (MEFs) [40] (these are established cell lines and therefore ethical approval is not required). Head and neck squamous cell carcinoma develops from the mucosal linings of the upper aerodigestive tract, comprising the nasal cavity and paranasal sinuses, the nasopharynx, the hypopharynx, larynx, trachea, and the oral cavity and oropharynx. Squamous cell carcinoma is the most frequent malignant tumor of the head and neck region and it was reported that head and neck squamous cell carcinoma is the sixth leading cancer by incidence worldwide [41]. A fibroblast is the most common type of cell found in connective tissue, secreting collagen proteins that are used to maintain a structural framework for many tissues (http://ghr.nlm.nih.gov/glossary=fibroblast). MEFs are often used as *feeder* cells in human embryonic stem cell research. In particular, MEFs have been utilized as a surrogate stem cell model for the postnatal bone marrow-derived stromal stem cells to study cell differentiation [42]. The study of MEF cells is important because it helps elucidate the molecular mechanisms underlying cellular immortalization, transformation, tumorigenesis; and can be used as a powerful tool to analyze the genetic regulation of these cellular processes [43].

Samples were prepared by a modified transmission electron microscopy method. Briefly, cells were fixed in solutions containing aldehydes, and sequentially treated with osmium/ferrocyanide, thiocarbohydrazide and osmium tetroxide. Before embedding in Epon 812 resin, samples were stained en bloc with uranyl acetate and lead aspartate to enhance backscatter electron signal especially from membranes. A plastic embedded sample was then sliced into hundreds of thin sections with 50 nm interval. The serially sectioned samples were collected on a piece of Si wafer, and they are subjected to taking photos in scanning electron microscope (SEM). This method easily avoids the charge-up, which is often a problem in using SEM, and allows us for acquiring high resolution images especially in the *z*-direction.

It should be noted about FIB-SEM that FIB and SEM are not separate imaging devices but the two components are combined as a whole to produce the images. The repetitive milling carried out by FIB and the image detection carried out by SEM in one vacuum chamber enable us to automatically obtain hundreds of serially-sectioned images of a cell. Otherwise, it is very laborious to obtain such imaging results. The specifications for producing the FIB-SEM images are as follows. The devise is Helios NanoLab 650 manufactured by FEI (http://www.fei.com/), applied with an acceleration voltage of 2kV and a working distance of 2.6 mm. The image resolution is of 2048 by 1768 pixels, whose size is of 18.5 by 18.5 nm. The magnification is 10kX. For this type of image data, the pixel size is more important than the magnification, which limits the image spatial resolution. The pixel size of 18.5 nm in our images is sufficiently small in comparison to the endoplasmic reticulum (ER) and other major organelles, because the luminal diameter of ER tubules is almost 100 nm.

## Largest Lyapunov exponents of images

A Lyapunov exponent is a real value which measures the dynamics of the evolution of trajectories in a phase space. The Lyapunov exponents of a phase space point (attractor) are referred to as the attractor’s Lyapunov spectrum. Lyapunov exponents quantify the chaos of a dynamical system by measuring its sensitive dependence on initial conditions or sensitivity to initial conditions [31]. A positive value of a Lyapunov exponent refers to the average rate at which predictability is unreliable for a bounded dynamical system that is chaotic (chaotic or strange attractor). A zero value indicates the dynamical system is of periodic limit cycle motion (nonchaotic or periodic attractor). A negative Lyapunov exponent implies that all trajectories move closer to each other or converge to a fixed point (nonchaotic or point attractor). Identification of the mechanism of dynamics underlying a system under study is useful because it provides insights into the uncertainty of the system so that appropriate modeling can be constructed for prototyping and simulating such a complex regime.

The calculation of the LLE is often used for analysis of time-series data, which is briefly presented here as the start of the development of the determination of the LLE for images. We now consider a discrete system with a 1-D map *x*_*k*+1_=*f*(*x*_*k*_) which evolves when it is started at two initial states *x*_0_ and (*x*_0_+*ε*_0_). The parameter *ε*_0_ takes a very small value to indicate the two initial states are very close to each other. The Lyapunov exponent is defined when the two trajectories are separated by a distance *ε*_*n*_ after *n* iterations of the map as follows.

(1) $$|\varepsilon_{n}|\approx |\varepsilon_{0}|\;e^{\mathrm{n\lambda}},$$

where *λ* is the Lyapunov exponent.

Taking the natural logarithm of both sides of Equation (1), the divergence of the two trajectories can be approximated as [31]

(2) $$\begin{array}{ll} \lambda \approx & \frac{1}{n}\ln\left|\frac{\varepsilon_{n}}{\varepsilon_{0}}\right|\\ = & \frac{1}{n}\ln\left|\frac{f^{n}(x_{0}+\varepsilon_{0})-f^{n}(x_{0})}{\varepsilon_{0}}\right|. \end{array}$$

If the interest is the study of the effects of very small perturbations, the limit of Equation (2) is taken as *ε*_0_→0, then the remaining term inside the logarithm is expanded using the chain rule which defines the derivative of the function *f* taken at *x*_0_:

(3) $$\begin{array}{ll} f^{n}(x_{0}+\varepsilon_{0})-f^{n}(x_{0})= & {\left(f^{n}\right)}^{\prime }(x_{0})\\ = & \overset{n-1}{\underset{i=0}{\prod }}f^{\prime }(x_{i}), \end{array}$$

which is the product of all the first derivatives or the rates of change for *i*=0 to *i*=*n*−1.

The back substitution of Equation (3) into Equation (2) gives

(4) $$\lambda \approx \frac{1}{n}\overset{n-1}{\underset{i=0}{\sum }}\ln|f^{\prime }(x_{i})|.$$

Finally, the limit of Equation (4) is taken as *n*→*∞*, giving

(5) $$\lambda =\underset{n\to \infty }{\lim}\left[\frac{1}{n}\overset{n-1}{\underset{i=0}{\sum }}\ln|\;f^{\prime }(x_{i})|\right].$$

For *K*-dimensional mappings, Equation (5) is extended to yield a spectrum of Lyapunov exponents arranged in a decreasing order:

$$\lambda_{1}\geq \lambda_{2}\geq \cdots \geq \lambda_{K},$$

where *λ*_1_ is known as the maximum or largest Lyapunov exponent (LLE) [33]. Equation (5) shows that the LLE is the average logarithm of the absolute values of *n* derivatives or local slopes (local divergences and/or convergences) taken over the entire attractor.

Having discussed before, because of the difficulty in the computation of the Lyapunov spectrum and the interest in estimating *λ*_1_ (LLE) which is the most significant indicator of chaos, the LLE estimate proposed by Rosenstein *et al.*[34] is applied in this study and works as follows.

Let **X**=(*x*_1_*x*_2_ … *x*_*N*_) be an original time series whose reconstructed trajectory can be represented as **X**^∗^=(**x**_1_**x**_2_ … **x**_*M*_)^*T*^ where **x**_*i*_=(*x*_*i*_*x*_*i*+*δ*_ … *x*_*i*+(*J*−1)*δ*_), and *M*=*N*−[ *δ*(*J*−1)] with given values for the lag *δ* and the embedding dimension *J*. The reconstructed phase space can be expressed in a matrix form as

(6) $$\text{X}={\left({\text{X}}_{1},{\text{X}}_{2},\ldots ,{\text{X}}_{M}\right)}^{T},$$

where **X** is a matrix of size *M*×*J*, *M*=*N*−(*J*−1)*δ*, and **X**_*i*_=(*x*_*i*_,…,*x*_*i*+(*J*−1)*δ*_) that is the state of the system at discrete time *i*.

The initial distance from the *j*th point to its nearest neighbor, denoted as *d*_*j*_(0), is defined using the Euclidean norm as

(7) $$d_{j}(0)=\underset{{\text{X}}_{j^{*}}}{\min}||{\text{X}}_{j}-{\text{X}}_{j^{*}}||,$$

where |*j*−*j*^∗^|>*MP*, and *MP* is the mean period which is the reciprocal of the mean frequency of the power spectrum.

The basic idea is that the LLE (*λ*_1_) for a dynamical system can be defined as [34]

(8) $$d(t)=c\;e^{\lambda_{1}t},$$

where *d*(*t*) is the average divergence of two randomly chosen initial conditions at time *t*, and *c* is a constant that normalizes the initial separation between neighboring points.

By the definition given in Equation (8), the *jth* pair of nearest neighbors can be assumed to diverge at a rate measured by *λ*_1_ as follows:

(9) $$d_{j}(i)\approx c_{j}\;e^{\lambda_{1}(\mathrm{i\Delta t})},$$

where *d*_*j*_(*i*) is the distance between the *jth* pair of nearest neighbors after *i* discrete-time steps which is *iΔt*, *Δt* is the sampling period of the time series, and *c*_*j*_ is the initial separation between two neighboring points.

Taking the logarithm of both sides of Equation (9), giving

(10) $$\ln[\;d_{j}(i)]\approx \lambda_{1}(\mathrm{i\Delta t})+\ln(c_{j}),$$

where $d_{j}(i)=||{\text{X}}_{j}(i)-{\text{X}}_{j^{*}}(i)||$. Equation (10) gives a set of approximately parallel curves, one for each *j* (*j*=1,…,*M*).

If these curves are approximately linear, their slopes represent the LLE (*λ*_1_). The LLE can be computed as the slope of a straight-line fit to the average logarithmic divergence curve defined by

(11) $$s(i)=\frac{1}{\mathrm{i\Delta t}}<\ln[\;d_{j}(i)]{>}_{j},$$

where <·>_*j*_ denotes the average over all values of *j*, which is essential to accurately estimate *λ*_1_ using a small dataset [34]. More detailed derivations of Eqs. 1 - 11 can be found in [31,34].

Using the same principle of the Rosenstein’s approach described above and taking advantage of the image structure where each row or column of an image can be viewed as a trajectory, an approximate procedure for measuring the sensitivity to initial conditions is designed.

The concatenations of the image rows (horizontal) and columns (vertical) are only two representative orientations of the spatial distribution of an image. However, such horizontal and vertical orientations have been found to be a good approximation to capture the spatial configuration of a two-dimensional image and to take into account the effect of an anisotropic structure [44].

The procedure works on a basis of the random selection of two nearest paths of pixels whose average rate of convergence or divergence in a phase space is to be measured to determine its state of disorder. Here, the phase space is an image of two dimensions. Sensitivity to initial conditions in our present study implies that each pixel point in an image space is arbitrarily closely approximated by other pixel points with significantly different future trajectories of image intensity values. Consider an image space in a matrix form as follows:

$$I=\left[\begin{array}{cccc} I_{11} & I_{12} & \cdots & I_{1N}\\ \cdot & \cdot & \cdots & \cdot \\ \cdot & \cdot & \cdots & \cdot \\ \cdot & \cdot & \cdots & \cdot \\ I_{M1} & I_{M2} & \cdots & I_{\text{MN}} \end{array}\right],$$

where *I*_*ij*_ is the image intensity at location (*i*,*j*) or the mean intensity value of a subimage of size *r*×*r* (for purpose of faster computation), whose top-left (TL), top-right (TR), bottom-left (BL), and bottom-right (BR) coordinates are defined as [ *TL*(*i*,*j*),*TR*(*i*,*j*),*BL*(*i*,*j*),*BR*(*i*,*j*)]=[ (*i*,*j*),(*i*,*j*×*r*),(*i*×*r*,*j*),(*i*×*r*,*j*×*r*)].

The nearest neighbor of pixel *I*_*ij*_, at spatial step *k*, can be determined by calculating the absolute differences between pixel *I*_*ij*_ and all other pixels row-wise or column-wise, respectively, such that

(12) $$\delta_{\text{ij}}^{R}(k)=\underset{p}{\min}(|I_{\text{ij}}-I_{\text{pj}}|),\;i\neq p,\;p=1,\ldots ,M,$$

where $\delta_{\text{ij}}^{R}(k)$ stands for the distance between pixel *I*_*ij*_ and its nearest-neighbor row pixel *I*_*pj*_, at *k* iterative step, or

(13) $$\delta_{\text{ij}}^{C}(k)=\underset{q}{\min}(I_{\text{ij}}-I_{\text{iq}}),\;j\neq q,\;q=1,\ldots ,N,$$

where $\delta_{\text{ij}}^{C}(k)$ stands for the distance between pixel *I*_*ij*_ and its nearest-neighbor column pixel *I*_*iq*_ measured at *k* iterative step.

Being analogous to the Rosenstein’s procedure, the average logarithmic divergence or convergence over all values of pixel points *I*_*ij*_, at spatial iterative step *k*, is defined row-wise or column-wise, respectively, by

(14) $$d_{R}(k)=\ln\left[\frac{1}{M}\overset{M}{\underset{i=1}{\sum }}\overset{R}{\underset{\text{ij}}{\delta }}(k)\right],$$

and

(15) $$d_{C}(k)=\ln\left[\frac{1}{N}\overset{N}{\underset{j=1}{\sum }}\overset{C}{\underset{\text{ij}}{\delta }}(k)\right].$$

Finally, the so-called LLE of the image can be determined by calculating the slope of the linear relationship of the plot of *d*_*R*_(*k*) or *d*_*C*_(*k*) versus *k*.

## Regularity dimension of images

Let **x**={*x*_1_,…,*x*_*N*_}, and *Q*_*m*_ be the set of all subsequences of length *m* in **x**: *Q*_*m*_={**x**_1*m*_,…,**x**_(*N*−*m*+1)*m*_}, where **x**_*im*_={*x*_*i*_,…,*x*_*i*+*m*−1_}. It is said that **x**_*im*_ and **x**_*jm*_ are similar if and only if

(16) $$|x_{i+k}-x_{j+k}|<r,\forall k,0\leq k<m,$$

where *r* is a threshold for similarity.

The probability of patterns of length *m* that are similar to the pattern of the same length that begins at *i* is

(17) $$C_{\text{im}}(r)=\frac{K_{\text{im}}(r)}{N-m+1},$$

where *K*_*im*_(*r*) is the number of subsequences in *Q*_*m*_ that are similar to **x**_*im*_.

The total average probability *C*_*im*_(*r*) for all *i*, *i*=1,…,*N*−*m*+1, is

(18) $$C_{m}(r)=\frac{1}{N-m+1}\overset{N-m+1}{\underset{i=1}{\sum }}C_{\text{im}}(r).$$

ApEn, given length *m* and tolerance value *r*, can now be readily computed by

(19) $$\text{ApEn}(m,r)=\log\left[\frac{C_{m}(r)}{C_{m+1}(r)}\right].$$

To avoid bias in self-matching encountered in ApEn, SampEn works in a slightly different way by defining **x**_*im*_ and **x**_*jm*_ are similar if and only if

(20) $$|x_{i+k}-x_{j+k}|<r,\forall k,0\leq k<m,i\neq \mathrm{j.}$$

Let *L*_*m*_={**x**_1*m*_,…,**x**_(*N*−*m*−1)*m*_}, the probability of patterns of length *m* that are similar to the pattern of the same length that begins at *i* is

(21) $$B_{\text{im}}(r)=\frac{J_{\text{im}}(r)}{N-m-1},$$

where *J*_*im*_(*r*) is the number of subsequences in *L*_*m*_ that are similar to **x**_*im*_.

The total average probability *B*_*im*_(*r*) for all *i*, *i*=1,…,*N*−*m*, is

(22) $$B_{m}(r)=\frac{1}{N-m}\overset{N-m}{\underset{i=1}{\sum }}B_{\text{im}}(r).$$

Finally, the value of SampEn, given *m* and *r*, can be calculated by the following equation:

(23) $$\text{SampEn}(m,r)=\log\left[\frac{B_{m}(r)}{B_{m+1}(r)}\right].$$

The regularity dimension (RD) [37] has been introduced to overcome the problem of selections of the two critical parameters *m* and *r* of ApEn and SampEn. The concept of the RD is based on the general rule of the exponent dimension defined as

(24) $$\Delta \propto s^{-D},$$

where *Δ*, ∝, *s*, and *D* stand for the number of increments, proportional to, scale size, and exponent dimension, respectively.

Based on Eq. (24), the exponent dimension can be obtained as

(25) $$D\propto \frac{\ln(\Delta )}{\ln(1/s)}.$$

It has been known that information increases with decreasing size of the lag space [30] such as *m* in this case. In other words, the information is approximately proportional to the log of 1/*m*. From now on information *I* is used to refer to SampEn. A straight line of the semilog axes is therefore expected in a plot of 1/*m* versus *I*, and the mathematical relation is a logarithmic equation. Because the relation is a straight line, it has the following form:

(26) $$I_{m}=a+D_{m}\ln(1/m),$$

where *I*_*m*_ is SampEn denoting the information subject to *m*, *a* is a constant which is the intercept, and *D*_*m*_ is the slope of the straight line, which is also the regularity dimension.

Rearrangement of (26) to solve for *D*_*m*_ yields

(27) $$D_{m}=\frac{I_{m}}{\ln(1/m)}-\frac{a}{\ln(1/m)}.$$

Setting a limit term on *m*, which indicates that the relation does not hold for very large *m*, gives

(28) $$D_{m}=\underset{m\to 0}{\lim}\frac{I_{m}}{\ln(1/m)}-\frac{a}{\ln(1/m)}.$$

When *m* gets smaller and smaller, 1/*m* and its logarithm becomes larger and larger. Since *a* is a constant, the term $\frac{a}{\ln(1/m)}$ approaches zero in the limit of *m* approaching zero, and therefore this term becomes negligibly small. Thus, (28) can be simplified as

(29) $$D_{m}=\underset{m\to 0}{\lim}\frac{I_{m}}{\ln(1/m)}.$$

It can be noted from (29) that the regularity dimension *D*_*m*_ measures the rate of change of signal regularity/predictability with respect to ln(1/*m*), it is the rate at which the entropy of a dynamical system is gained with increasing resolution (decreasing length *m*).

Alternatively, increasing the value of *r*, which is the tolerance of similarity, decreases the information in the sense that no information is gained when most subsequences are considered to be similar (signal is highly predictable or has low complexity). Being analogous to the relation of a straight line developed for *D*_*m*_, the regularity dimension *D*_*r*_ can be obtained using the expression for ApEn in terms of *r* as follows.

(30) $$I_{r}=a+D_{r}\ln(1/r),$$

where *I*_*r*_ is ApEn subjected to *r*, *a* is a constant which is the intercept, and *D*_*r*_ is the slope of the straight line, which is the regularity dimension.

Rearrange Equation (30), we can define *D*_*r*_ as

(31) $$D_{r}=\frac{I_{r}}{\ln(1/r)}-\frac{a}{\ln(1/r)}.$$

To indicate the relation that does not apply to very large *r*, a limit term is inserted on *r*, giving

(32) $$D_{r}=\underset{r\to 0}{\lim}\frac{I_{r}}{\ln(1/r)}-\frac{a}{\ln(1/r)}.$$

Removing the negligible term $\frac{a}{\ln(1/r)}$ in the limit of *r* approaching zero gives

(33) $$D_{r}=\underset{r\to 0}{\lim}\frac{I_{r}}{\ln(1/r)}.$$

The above procedure is designed for the analysis of time-series data. A feasible way for the estimate of the RD in images is to concatenate the pixel values in either row-wise, column-wise, or diagonal-wise to transform the 2-dimensional image into a sequence of image intensities, which is then ready for application of the described computation of the RD in terms of either *r* or *m*. For the RD computation, the generation of the time series of the image in either row-wise, column-wise, or diagonal-wise direction is only a representative orientation of the spatial distribution of an image. The generation of the time series of the image in any orientation may be considered. However, in many applications, horizontal (row-wise) and vertical (column-wise) orientations have been found to be a good approximation to capture the spatial configuration of a two-dimensional image and to take into account the effect of an anisotropic structure [44-46].

## Results

**Figure 1:**
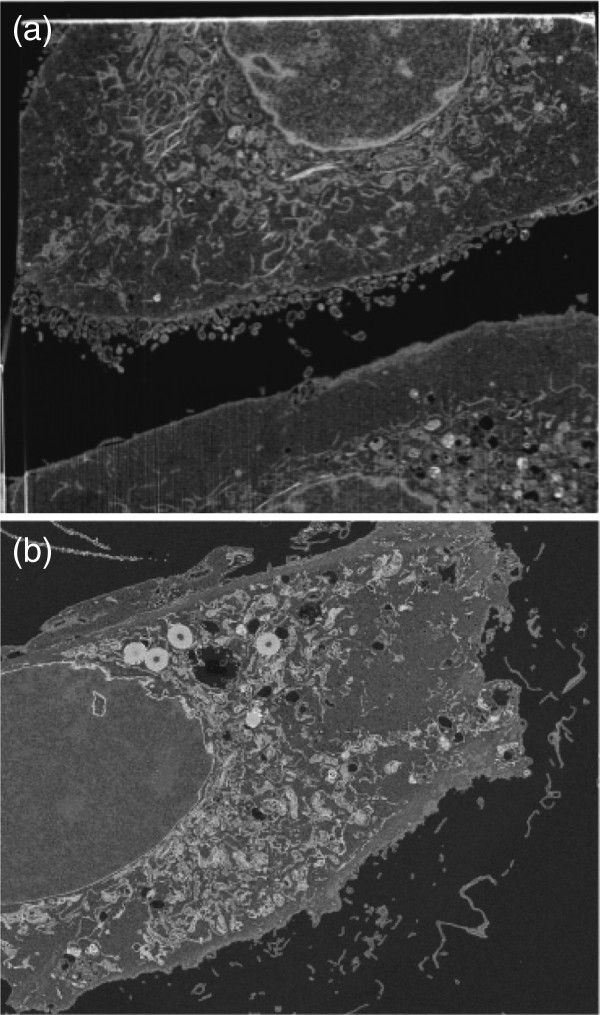
Typical partial images of (cancer) SCC-61 (a) and (normal) MEF (b) cells, of which organelles are revealed by the combination of scanning electron microscopy (SEM) and focused ion beam (FIB) technology (part of the cell, including the nucleus, membrane and organelles, can be seen on the upper half of SCC-61).

**Figure 2:**
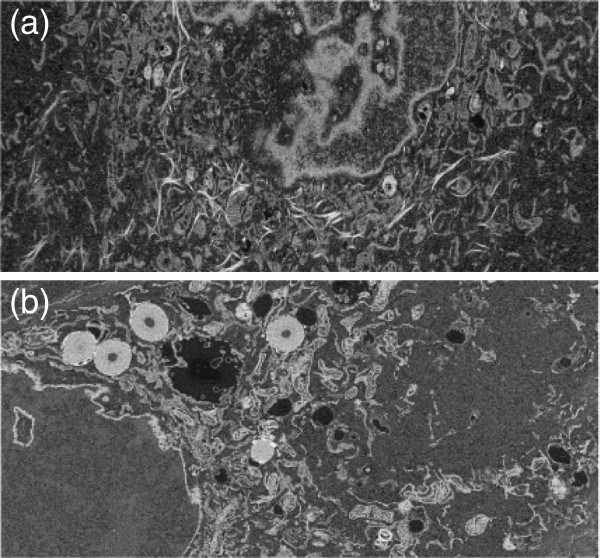
Images of the intracellular space of SCC-61 (a) and MEF (b), where the maximum regions containing the organelles were extracted from the corresponding original images.

Figure 1 shows two typical FIB-SEM images of SCC-61 (cancer) and MEF (normal) cells, where the organelles of each cell can be visually observed in the intracellular space. Figure 2 shows the extracted intracellular space segments of FIB-SEM images of SCC-61 (Figure 2 (a)) and MEF (Figure 2 (b)) cells. Rosenstein’s method and the proposed approximate method for estimating LLEs were applied to study the spatial chaos of the intracellular space of the cells. Twenty scans of the FIB-SEM images of the cancer single cell, and sixty FIB-SEM images of the intracellular space of the normal cell of the MEF were used to implement the Rosenstein’s method for estimating the LLE. The image intensities were row-wise and column-wise concatenated to generate row-wise and column-wise time-series data of the image, respectively.

**Figure 3:**
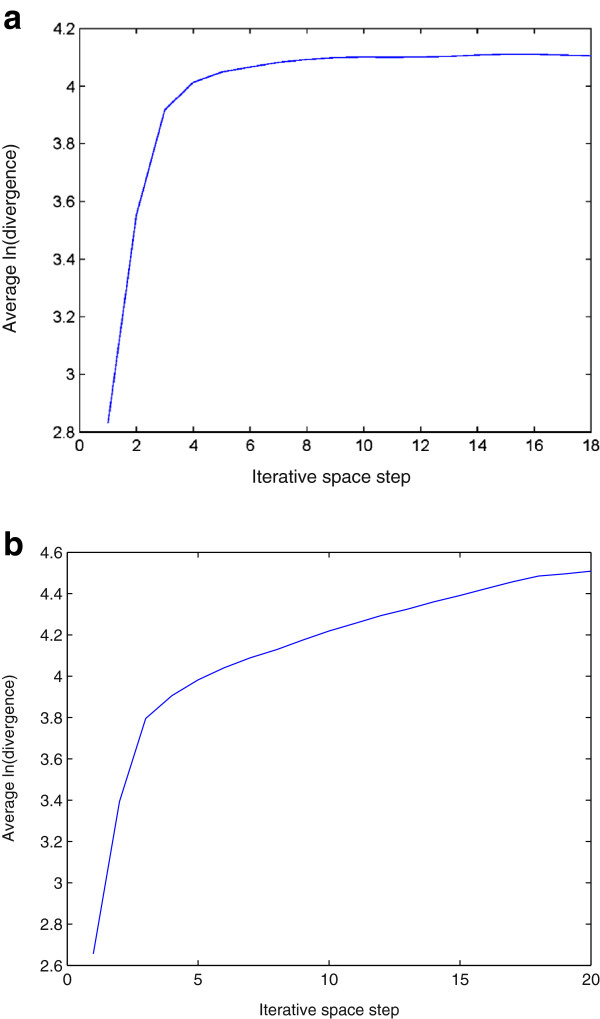
Logarithmic divergence versus iterative steps obtained by Rosenstein’s method using Eq. (11) with row-wise time-series data generated from intracellular space of SCC-61 (a) and MEF (b), where the LLE of each plot was estimated by calculating the slope of the least-squares straight line fit.

**Figure 4:**
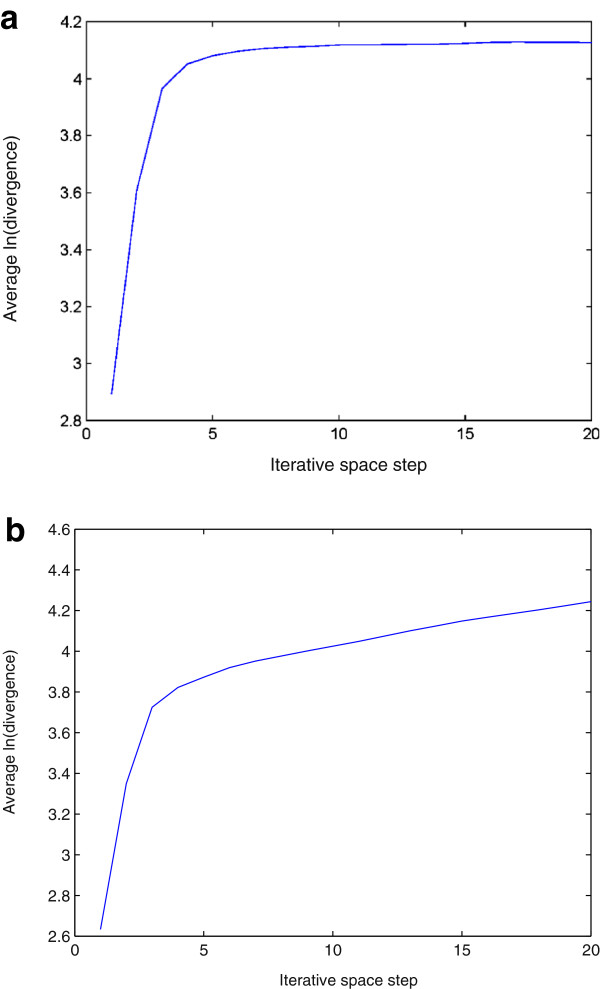
Logarithmic divergence versus iterative steps obtained by Rosenstein’s method using Eq. (11) with column-wise time-series data generated from intracellular space of SCC-61 (a) and MEF (b), where the LLE of each plot was estimated by calculating the slope of the least-squares straight line fit.

**Figure 5:**
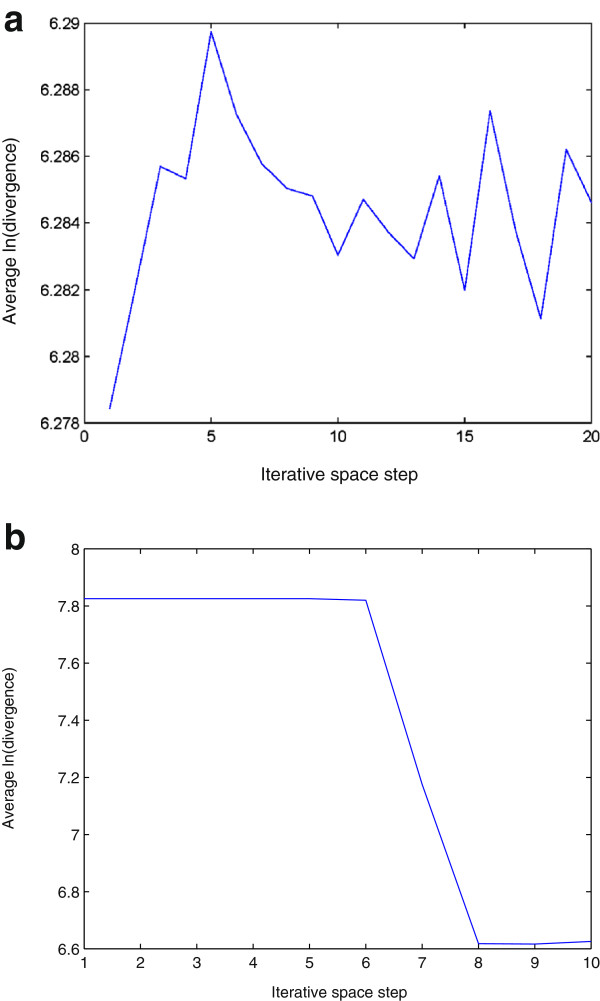
Logarithmic divergence versus iterative steps obtained by the proposed approximate LLE method using Eq. (14) with row-wise time-series data generated from intracellular space of SCC-61 (a) and MEF (b), where the LLE of each plot was estimated by calculating the slope of the least-squares straight line fit.

**Figure 6:**
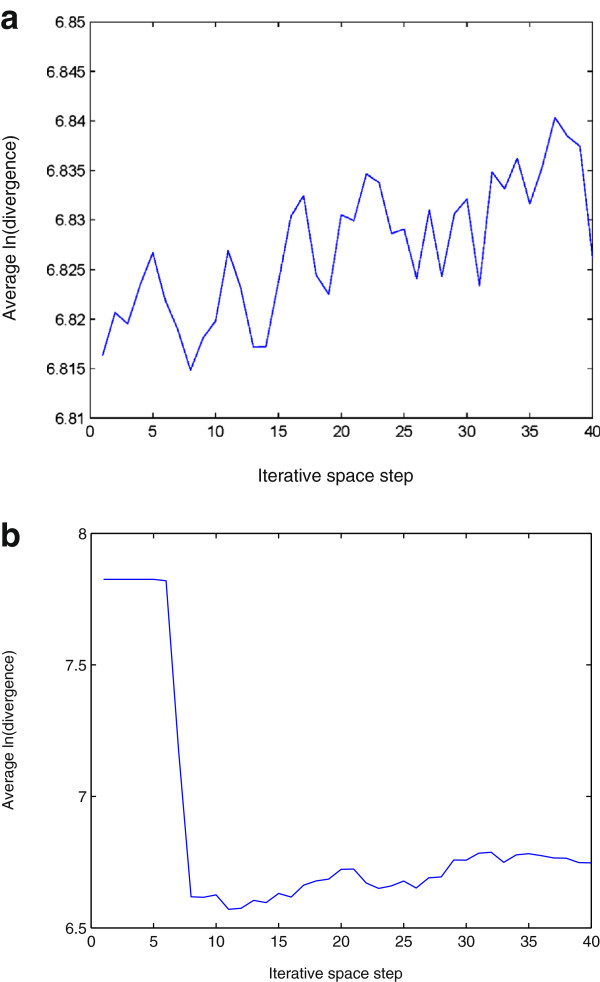
Logarithmic divergence versus iterative steps obtained by the proposed approximate LLE method using Eq. (15) with column-wise time-series data generated from intracellular space of SCC-61 (a) and MEF (b), where the LLE of each plot was estimated by calculating the slope of the least-squares straight line fit.

The lag and the number of the embedding dimensions were selected to be 1 and 3 for the phase-space reconstruction, respectively. For the calculation of the approximate LLE expressed in Eqs. (14) and (15), a window of size 4×4 was used to obtain a mean intensity value. Figures 3 and 4 show the plots of the logarithmic divergence versus iterative steps of the row-wise and column-wise time-series data, respectively, of the two typical images shown in Figure 2, including only the organelles of the single cancer and normal cells. For the SCC-61, the straight segments of the slopes in both cases indicate positive LLEs (chaotic) where convergences are quickly reached after a few iterations. The same images of SCC-61 and MEF were used to test for chaos using the proposed approximate estimate of the LLE. Figures 5 and 6 show the plots of the logarithmic divergence versus iterative steps of the row-wise and column-wise time-series data of the two cell types, respectively. Table 1 shows the average LLEs of the intracellular space of the cancer cell SCC-61 and normal cell MEF using the Rosenstein’s and proposed (approximate) methods.

**Table 1:** LLEs (mean, variance) of the intracellular space of SCC-61 (cancer) and MEF (normal) cells based on different orientations for time-series generations (Rosenstein’s method results are obtained from Eq. (11), and approximate LLE method results are obtained from Eq. (14) for row-wise and Eq. (15) for column-wise)

**Method**	**Row-wise**	**Column-wise**
	**SCC-61**	
Rosenstein (long range)	(0.5432, 0.00021)	(0.5356, 0.00016)
Approximate (short range)	(0.0026, 0.00003)	(0.0004, 0.00001)
	MEF	
Rosenstein (long range)	(0.3800, 0.00032)	(0.3640, 0.00046)
Approximate (short range)	(0, 0.00002)	(0, 0.00001)

Regarding the complexity analysis, both SampEn and RD were applied to measure the predictability of the real intracellular space of the SCC-61 and MEF cells. Because of the selection of the similarity threshold *r* has been known to be much more critical than the sublength *m* used in SampEn, only the estimate of *D*_*r*_ was carried out in this experiment. The horizontal (*x*), vertical (*y*), and diagonal (*z*) intensity values of the images were extracted by concatenating the intensity values in the same direction to produce 3 time-series datasets. Values of *m* and *r* were chosen to be 3 and 0.3*σ* where *σ* is the standard deviation of the signal, respectively. For the calculation of the RD, the range of the tolerance of the similarity threshold *r* was specified from 0.1*σ* to 0.5*σ* with a step size of 0.025*σ*. The values of the SampEn and RD for the cancer and normal cell images are shown in Tables 2 and 3, respectively. Furthermore, we applied the window sizes of 3×3, 5×5 and 7×7 to average the pixel intensity to reduce noise in the images by replacing the value of each pixel by the average value inside the window centered at the pixel (low-pass filtering). The mean values of the LLE, SampEn and RD of the SCC-61 and MEF cells using the three different window sizes are shown in Table 4.

**Table 2:** SampEn (mean, variance) of the intracellular space of SCC-61 (cancer) and MEF (normal) cells using different orientations, obtained from Eq. (23)

**Horizontal**	**Vertical**	**Diagonal**
	**SCC-61**	
(0.810, 0.00046)	(0.795, 0.00038)	(0.837,0.00030)
	MEF	
(0.372, 0.00029)	(0.356, 0.00020)	(0.369,0.00027)

**Table 3:** Regularity dimensions (mean, variance) of the intracellular space of SCC-61 (cancer) and MEF (normal) cells using different orientations, obtained from Eq. (33)

**Horizontal**	**Vertical**	**Diagonal**
	**SCC-61**	
(0.141, 0.00002)	(0.135, 0.00001)	(0.241,0.00001)
	MEF	
(0.052, 0.00001)	(0.058, 0.00001)	(0.126,0.00001)

**Table 4:** **Mean values of LLE, SampEn, and RD of the intracellular space of SCC-61 and MEF cells using different window sizes and orientations (the values in brackets indicate the results obtained from the window sizes of *3×3*, *5×5*, and *7×7*, respectively)**

**SCC-61**			
	Row-wise	Column-wise	
LLE (long range)	(0.5432, 0.5430, 0.5427)	(0.5354, 0.5350, 0.5348)	
Approximate (short range)	(0.0025, 0.0024, 0.0022)	(0.0004, 0.0003, 0.0003)	
	Horizontal	Vertical	Diagonal
SampEn	(0.802, 0.798, 0.785)	(0.795, 0.797, 0.788)	(0.830, 0.832, 0.827)
RD	(0.141, 0.139, 0.138)	(0.135, 0.132, 0.132)	(0.241, 0.238, 0.235)
**MEF**			
	Row-wise	Column-wise	
LLE (long range)	(0.3800, 0.3766, 0.3762)	(0.3640, 0.3581, 0.3552)	
Approximate (short range)	(0, 0, 0)	(0, 0, 0)	
	Horizontal	Vertical	Diagonal
SampEn	(0.368, 0.363, 0.363)	(0.350, 0.346, 0.346)	(0.362, 0.354, 0.350)
RD	(0.049, 0.045, 0.043)	(0.050, 0.048, 0.045)	(0.125, 0.121, 0.118)

## Discussion

All positive LLEs shown in Table 1 suggest the chaotic behavior of the intracellular space of the cancer cell. Here the LLE is an important indicator of detecting and characterizing chaos produced from a spatial dynamical system of the real intracellular space. The LLEs in both rows and columns of the image space estimated by the Rosenstein’s method are very similar and higher than those by the approximate method of which row-wise LLE is higher than column-wise LLE. While the LLEs estimated by the Rosenstein’s method (long range) in both row-wise and column-wise directions of the intracellular space are similar, the approximate LLE (short range) in the row-wise direction is several times (6.5) higher than that in the column-wise direction. This observation suggests the change in the image intensity of the organelles along the horizontal direction of the cell is much more chaotic than that of the vertical direction of the cell.

Based on the nature of the data modeling, the LLEs obtained from the Rosenstein’s method (Table 1) can refer to the long-range behavior of the phase space by joining all the pixels into a single time series, whereas the LLEs obtained from the proposed method (Table 1) refer to the short-range behavior of the intracellular space by considering shorter paths along the vertical and horizontal directions of the image. Regarding the complexity measure, the diagonal content of the image yields the highest RD indicating the poorest predictability or most complexity of the signal in the diagonal orientation of the intracellular space. In this study, the RD is calculated in terms of the SampEn. For a fixed value of *r*, that is 0.3*σ*, the mean SampEn values of the cancer-cell signals in the horizontal (*x*), vertical (*y*) directions are similar and slightly lower than the SampEn value of the cancer cell in the diagonal (*z*) orientation (Table 2). Meanwhile, by allowing a range of values for the threshold *r* in the calculation of the SampEn, the RD values of the cancer-cell signals can be better distinguished between different spatial orientations where the RD in the *z* direction is as twice larger than those in the *x* and *y* directions (Table 3). This observation indicates the diagonal signal of the cancer cell is more complex than the others.

The LLE values of the normal MEF cell obtained from the Rosenstein’s method (long range) in both row-wise and column-wise orientations (Table 1) are about 1.5 times lower than those of the cancer cell SSC-61 (Table 1) previously discussed, indicating a much less magnitude of a chaotic behavior. Meanwhile, the LLE values of the MEF cell estimated by the approximate method (short range) (Table 1) in both orientations are zeros, suggesting the spatial distribution of the intracellular space of the normal cell is non-chaotic. The SampEn values of the normal cell (Table 2 are about half less than those of the cancer cell (Table 2). Once again, the use of the RD can help better distinguish the difference in the complexity between the intracellular signal in the *z* direction versus the signals in the *x* and *y* directions, where the RD value in the *z* direction is about twice larger than those in the *x* and *y* directions (Table 3). The magnitudes of the complexity in both *x* and *y* orientations of either the cancer or normal cell are found to be similar.

While the average logarithmic divergence of the cancer intracellular space in either row-wise (Figure 3) (a) or column-wise (Figure 4) (a) orientation using the Rosenstein’s method saturates at longer times since the system is bounded in phase space [34], the average logarithmic divergence of the cancer intracellular space in either row-wise (Figure 5 (a)) or column-wise (Figure 6 (a)) orientation using the approximate LLE method fluctuates along the spatial iterations. This is because the use of a smaller phase space modelled by Eqs. (14) and (15), where abrupt changes in the distances in terms of the image intensity between the nearest-neighbor pixels can be expected. Similar converegence and fluctuation of the average logarithmic divergence in either row-wise or column-wise orientation are found in the analysis of the normal intracellular space using the Rosenstein’s method (Figures 3 (b) and 4 (b)) and the approximate method (Figures 5 (b) and 6 (b)). The flat saturation at the beginning of the curves using the approximate method shown in Figures 5 (b) and 6 (b) can be easily observed, suggesting the non-chaotic behavior in the modeling of the intracellular space, where the value of the LLE is zero.

When a system has a positive LLE, there is an implication that small disturbances will give rise to exponential divergence. In other words, the LLE determines a notion of predictability for a dynamical system. In general, the LLE of the SCC-61 was found to be about 1.5 higher than that of the MEF. Based on the meaning of the LLE, the intracellular space of the SCC-61 is subject to a more spatially changing environment than that of the MEF. The LLE results of the two cell types also agree with the interpretation of the degree of the signal predictability or regularity obtained from the SampEn and RD methods, where the spatial distribution of the intracellular space of the MEF was found to be about half less complex than the SCC-61 in various spatial orientations. Beside the spatial dynamics of the intracellular space distribution, the intracellular complex systems were indirectly characterized in terms of texture complexity inherently existing in the images, where image edges play an important criterion, because image texture provides information about the spatial arrangement of color or intensities in an image or a selected region of an image [47]. Here, the texture information of the SCC-61 images appear to be more complex or richer (indicated by the magnitudes of the LLE, SampEn and RD) than that of the MEF images. Such an additional characterization can be useful for the classification of molecular [48,49] and biological tissue [50] images.

Regarding the image formation, the scanning process of the FIB-SEM images are different from laser scanning confocal microscopes in which, depending on the scanning mode, the pixel acquisition of the next line starts either in a reverse order relative to the preceding line, or in the same order but only after another reversal of the sweep direction [51]. The areas of the cell surfaces studied here were scanned in a raster fashion where the beam sweeps horizontally left-to-right at a steady rate, then blanks and rapidly moves back to the left, where it turns back on and sweeps out the next line; therefore the separation of pixels in either rows or columns of the image is consistent in the concatenated time-series. Regarding the reproducible statistics of the results; the maximum intracellular regions of all scans of the cells, which exclude the non-organelle background, were selected for the analysis of the spatial chaos and complexity. Such a selection of the intracellular space of each image scan of the cells guarantees the negligible variance of the results. By applying the low-pass filter with the window sizes of 3×3, 5×5 and 7×7 to the intracellular space images of the cancer and normal cells, the results obtained from the same procedures for the calculations of the LLE (long range and short range), SampEn and RD (Table 4) were not significantly different to about two decimal places from those without the averaging processes.

The potential exhibitions of complexity, short-range and long-range chaotic behaviors of the cancer intracellular space as suggested in this study can provide some insight into our modeling and simulation of the single cancer cell spatial parameters [39]. The intracellular organelles such as mitochondria and endoplasmic reticulum can act as obstacles for the protein diffusion. As an example, if the distribution of mitochondria is regular in line, their effect as obstacles will be smaller in comparison to the staggered distribution. Thus, the distribution of mitochondria will change the oscillation pattern of the protein complex if the number of mitochondria is the same. The chaotic distribution of intracellular organelles can dramatically alter the oscillation pattern of the protein diffusion. In summary, given that the relations and distributions between cell organelles (nucleus, ribosome, vesicle, rough endoplasmic reticulum, Golgi apparatus, cytoskeleton smooth endoplasmic reticulum, mitochondria, vacuole, cytosol, lysosome, centrioles within centrosome, cell membrane) can be complex, the simulation can be more accurate over several local regions rather than the whole intracellular space as the short-range LLE estimated by the proposed method suggests negligible rate of the loss of predictability.

The cells used in the current analysis were in the interphase. However, it is known that cells in the cultivating dish change due to their life cycle, and this change has a substantial effect on their structure that includes their appearance revealed in the image. These differences are extensive and dependent on the cell type. Therefore, the state of cells at the time of fixation should be specified and considered in the interpretation of results. Such a comprehensive study is an important target of our near future research. In order to define the life cycle, we should fix and immunolabel cells, and measure their fluorescence. Alternatively, we can use fluorescence ubiquitination cell cycle indicator [52] that would allow us to follow cell division within a cell population. In this paper, we intended to show a possibility to characterize cells using our proposed methods

## Conclusion

We have presented the application of the largest Lyapunov exponent to investigate the possible chaotic behavior of typical cancer and normal intracellular signals obtained from FIB-SEM imaging system. The positive values of the LLE of the cancer cell, which are twice larger than those of the normal cell, indicate some degree of chaos exhibiting in the spatial distribution of the intracellular space of the cancer. The short-range LLE values of the normal cell are zeros in both *x* and *y* directions refers to the non-chaotic distribution of the organelles in the intracellular space under a specific image structure model. Appropriate numerical methods for the determination of largest Lyapunov exponents are important for accurate quantification of the chaotic behavior of the problem under study. We have further presented the application of nonlinear dynamical methods to investigate the complexity of cancer intracellular signals on FIB-SEM images and can distinguish the difference in the complexity between cancer-cell and normal-cell models. The relatively high values of the SampEn and RD found in the intracellular space of the cancer cell suggest the potential complexity of the cancer cellular information.

It should be noted that, due to the limited experimental materials, the results presented here are of illustrations of the capabilities of the proposed image analysis and not as a suggestion of the proof on differences between cancer and non-cancer cells. Nevertheless, the initial findings of this study are promising for providing an insight into a challenging problem of identification of an underlying mechanism of cancer imaging, which can be used for constructing appropriate types of modeling, simulation and control of cancer.

## Competing interests

The authors declare that they have no competing interests.

## Authors’ contributions

Both TDP and KI conceived the study. TDP designed the mathematical models, developed the computational implementations, and drafted the manuscript. Both authors read and approved the final manuscript.
